# Sensibility of the Teeth Explained on Hydrostatic Principles

**Published:** 1850-09

**Authors:** J. Neill

**Affiliations:** Demonstrator of Anatomy in the University of Pennsylvania


### RECORD OF MEDICAL SCIENCE.

### ANATOMY AND PHYSIOLOGY

*Sensibility of the teeth explained on Hydrostatic principles.* By J. NEILL, M. D., Demonstrator of Anatomy in the University of Penn.—Dr. Neill called the attention of the College to the fact that no satisfactory explanation had yet been offered of the manner in which external impressions made upon the teeth are conveyed to their nervous pulp within. There is no difficulty in understanding the manner in which the nerves of the eye, of the olfactory organs, of the tongue, the skin, &c., are reached by the impressions for the reception and transmission of which they are respectively designed. But in the teeth the nerves are enclosed in a bony case ; and yet experience teaches us that impressions made upon the teeth do reach the nerve within, and are conveyed through it to the nervous centre. How then is it that heat, cold, acids, or pressure when applied to the external surface of the teeth affect the nervous pulp within them? Some may suppose that the impression is transmitted to the nerve through the gums. It is doubtful whether the gums in their ordinary healthy condition possess any very great amount of nervous sensibility, at any rate this explanation is untenable. The old doctrine of increased sensibility of the teeth from inflammation has passed away ; the teeth have neither blood-vessels nor nerves, and consequently cannot become the seat of inflammation. Dr. Neill believed that the transmission of impressions through the teeth may be explained by a reference to the anatomical structure. The parietes of the tooth are not perfectly solid ; even the enamel is composed of a congeries of hexagonal prisms, which are not in perfect juxtaposition. This may be shown by the microscope. The bony portion beneath the enamel is even less solid, it is somewhat allied to bone, and consists of a number of minute hollow tubules, which pass from the circumference to the centre of the teeth, with the internal cavity of which they communicate ; these tubules are filled with a fluid secreted by the pulp. Now if the tubules, thus filled, pass from the circumference to the interior of the teeth, may not pressure applied without, by compressing the enamel and the fluid of the tubes, affect the nervous pulp within, by subjecting the latter to a species of hydrostatic pressure, the amount of which can be measured ? Whatever reduces the thickness of the enamel, or uncovers any portion of the dentine increases the painful impressions caused by external pressure. Thus acid, by removing the mucus by which the teeth are ordinarily covered and perhaps a portion of the enamel, increases the sensibility to external impressions. When the teeth have been affected by acids, no disagreeable sensation is experienced until pressure is made upon them. Mr. Nasmyth, it is true, denies the tubular structure of the dentine, but it is admitted by all other writers on the anatomy of the teeth. Dr. Neill presented a specimen which would, he remarked, under the microscope, show very clearly the tubular structure of the dentine. Of the conveyance of impressions to nervous expansions by hydrostatic pressure, we have an example in the ear. Dr. Neill supposed that all those substances which are known to relieve tooth ache, when applied to the affected tooth, acted by coagulating the fluid of the tubuli, and thus preventing hydrostatic pressure.—*Transactions o*[c] *the College of Physicians.*

